# Rate–Distortion Limits for Task-Oriented Compression with Side Information

**DOI:** 10.3390/e28060593

**Published:** 2026-05-26

**Authors:** Tao Guo, Zhangyao Song, Huihui Wu, Yang Li

**Affiliations:** 1 School of Cyber Science and Engineering, Southeast University, Nanjing 210096, China; taoguo@seu.edu.cn (T.G.); zysong@seu.edu.cn (Z.S.); 2 Ningbo Institute of Digital Twin, Eastern Institute of Technology, Ningbo 315200, China; 3 Ningbo Key Laboratory of Spatial Intelligence and Digital Derivative, Ningbo 315200, China; 4 School of Economics, University of Nottingham Ningbo China, Ningbo 315100, China; yang.li@nottingham.edu.cn

**Keywords:** semantic compression, rate–distortion function, side information

## Abstract

This paper analyzes the semantic rate–distortion problem motivated by task-oriented data compression with side information. The semantic information related to a task is not directly accessible to the encoder but implicitly impacts the observations through a joint probability distribution. The decoder aims to simultaneously recover the observation and infer the semantic information under certain distortion constraints. Notably, this paper advances the related research by involving side information and the observation of two semantic segments at both the encoder and decoder, which significantly complicates the theoretic analysis. We establish the information-theoretic limits for the tradeoff between compression rates and distortions by fully characterizing the rate–distortion function. Additionally, we explicitly derive the corresponding rate–distortion functions under specific Markov conditions for two scenarios: (i) the task is a binary classification of an integer observation as even and odd; and (ii) Gaussian-correlated task and observation. Furthermore, we validate the information-theoretic analysis by conducting a classification-oriented lossy image compression based on deep learning. The results are consistent with theoretical expectations, demonstrating the effectiveness of side information on both distortion and classification accuracy and the rationality of semantic segmentation.

## 1. Introduction

It is known that lossy source coding under given fidelity criterion was introduced in Shannon’s landmark paper [1], and the *rate–distortion function* was further proposed in [2], characterizing the optimal tradeoff between compression rates and distortion measurements from the perspective of mutual information. However, the classical lossy compression aims to reconstruct only the original source under certain distortion constraints, which is not aligned with the efficiency requirements of intelligent systems in the current AI era. Taking the traffic violation detection as an example, one would expect that a single-step compression algorithm optimized for the detection accuracy would be significantly more efficient than the straightforward recover-and-detect approach, where the video/image is first decompressed before detection. Indeed, this has been validated in related studies, e.g., [3,4,5].

Therefore, task-oriented or semantic compression [6,7,8,9] tailored for a specific task (e.g., video inference, decision making, classification, etc.) is gaining increasing attention. As shown in [3,4,5], the compression efficiency is improved by recovering only the most relevant semantic information corresponding to a given task rather than the entire source as in classical Shannon rate–distortion setups. Accordingly, in order to build its information-theoretic foundation, the classical rate–distortion theory has to be revisited considering the emerging semantic constraints.

Recently, the classical indirect source coding problem [10] was revisited from the semantic point of view, establishing the theoretical limits for point-to-point semantic compression in [11]. Specifically, they considered the lossy compression of sources (S,X), where *S* is interpreted as intrinsic semantic information and *X* is the corresponding extrinsic observation. Leveraging the classical indirect rate–distortion theory, they characterized the semantic rate–distortion function, when the decoder reconstructs both $\hat{S}$ and $\hat{X}$ with two separate distortion constraints. Subsequently, the authors of [12] investigated the impacts of different distortion measures, especially the context-dependent distortion in semantic compression. Moreover, a variant of the Blahut–Arimoto algorithm was developed to compute the point-to-point semantic rate–distortion functions for discrete sources [12]. Most recently, the semantic compression with rate–distortion, perception and classification constraints was studied [13] from the perspective of information bottleneck. In addition, the semantic lossy compression has been extended to the joint source-channel coding scenario [14].

Nevertheless, the aforementioned information-theoretic analysis solely concentrates on the point-to-point semantic compression. Tracing the footsteps of classical rate–distortion theory research, the introduction of side information could greatly improve compression efficiency. The rate–distortion function was investigated when side information is available at the encoder or/and decoder; see [15,16,17,18] and the references therein.

Specifically, in the case where the side information is only available at the encoder, then no benefit could be achieved. In contrast, when side information is only accessible at the decoder, the corresponding rate–distortion function was considered by Wyner and Ziv in [17] with its extensions being discussed in [18]. Finally, if both the encoder and decoder have access to the same side information, the optimal tradeoff is called *conditional rate–distortion function*, which was given by [15,16]. In addition, Slepian and Wolf [19] solved a more general problem of distributed compression. In practice, lossy source coding with side information has been applied in video compression standards such as H.264 and HEVC, as well as in learning-based multi-view video compression [20].

Given the pivotal role of side information in classical rate–distortion theory, it is natural to wonder whether it has a positive impact on semantic lossy compression. This paper affirms that it does by introducing side information *Y* to both the encoder and decoder and fully characterizing the corresponding rate–distortion function. In doing so, this paper also advances the prior point-to-point semantic rate–distortion theory [11,12,13] into the distributed case.

Moreover, considering the practical scenario where the observation (e.g., images in the street view house numbers (SVHN) dataset) typically contains different semantic information (e.g., numbers, colors, background elements such as walls) that indicates varying levels of relevance for a given task (e.g., the numbers are more relevant than the background for classification tasks), this paper further generalizes the theoretical analysis by partitioning the observation as a pair of variables $\left(X_{1},X_{2}\right)$ to model different relevance levels. Without loss of generality, $X_{1}$ denotes the most relevant semantic in the observation and $X_{2}$ represents the remaining. In practical applications such as autonomous driving and intelligent surveillance, although downstream machine vision tasks primarily rely on the highly relevant features in $X_{1}$, recovering the background or appearance segment $X_{2}$ remains crucial for human operators to maintain situational awareness. Under resource-constrained channels, a hybrid human–machine vision system can allocate fewer bits to $X_{2}$ (by allowing a larger distortion $D_{2}$) while ensuring high fidelity for $X_{1}$ and high accuracy for task inference. We point out that [21] has already investigated the varying relevance of different objects in an image for downstream machine vision tasks by proposing a semantically disentangled image coding framework, which necessitates the information-theoretic observation model in this paper.

In addition to the theoretical semantic rate–distortion limits, we implement a classification-oriented image compression pipeline using autoencoders. The extensive experimental results confirm the positive impact of side information on both classification accuracy and distortion, and they affirm the rationality of the generalized observation $\left(X_{1},X_{2}\right)$, which aligns with the derived theoretical analysis.

To summarize, this paper primarily advances the information-theoretic analysis of semantic lossy compression with side information, particularly by modeling the semantic source as $\left(S,X_{1},X_{2},Y\right)$, which results in a notably non-trivial characterization of rate–distortion functions. The main contributions are listed as follows:

- We formulate a theoretical semantic rate–distortion framework involving side information *Y* and the observation of two semantic segments $\left(X_{1},X_{2}\right)$. The corresponding optimal rate–distortion function is fully characterized, which is followed by an exploration of some key properties.
- We reveal that the separate compression of $X_{1}$ and $X_{2}$ is optimal if they are conditionally independent given the side information *Y*.
- The rate–distortion functions are explicitly discussed for cases when the following apply: (i) $\left(S,X_{2},Y\right)$ are binary sources and $X_{1}$ is an integer source (i.e., an odd-even classification of integers) and (ii) $\left(S,X_{1},X_{2},Y\right)$ are all Gaussian sources.
- An autoencoder-based image compression scheme is implemented for a classification task. The experimental results substantiate the positive effect of side information on both distortion and classification accuracy, which is in line with the theoretical analysis.

The remainder of this paper is organized as follows. We first formulate the problem and present some preliminary results in Section 2. In Section 3, we characterize the rate–distortion function and some useful properties. Numerical evaluations of the rate–distortion functions for binary, integer, and Gaussian sources are presented in Section 4.1 and Section 4.2, respectively. We conduct experiments on a deep learning-based image compression scheme for a classification task in Section 5. Finally, the paper is concluded in Section 6. Some essential proofs can be found in the appendices.

## 2. Problem Formulation and Preliminaries

### 2.1. Problem Formulation

**Figure 1:**
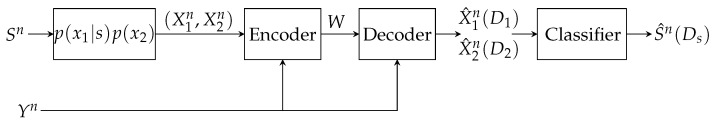
Illustration of system model with side information.

The proposed theoretical semantic compression model is illustrated in Figure 1, where the side information is known at both the encoder and decoder sides.

The problem is formally defined as follows. A collection of *discrete memoryless sources* (DMS) is described by generic random variables $\left(S,X_{1},X_{2},Y\right)$ taking values in finite alphabets $S\times X_{1}\times X_{2}\times Y$ according to probability distribution $p\left(x_{1},x_{2},y\right)p\left(s|x_{1}\right)$. In particular, this indicates the Markov chain $S-X_{1}-\left(X_{2},Y\right)$. We interpret *S* as a latent variable, i.e., the intrinsic semantic information (e.g., the state of a cognitive system), which is not observable by the encoder. We assume that the extrinsic observation consists of two parts:

- $X_{1}$ dynamically varies according to the semantic information *S*, which captures the most relevant segment, e.g., the cars and red lights in a frame showing a traffic violation;
- $X_{2}$ is the remaining “appearance” of the observation, e.g., the remaining elements in the frame capturing the violation.

We interpret *Y* as the side information that improves compression efficiency such as previous frames in a video. For length-*n* source sequences, $\left(S^{n},X_{1}^{n},X_{2}^{n},Y^{n}\right)$, the encoder has access to only the observed ones $\left(X_{1}^{n},X_{2}^{n},Y^{n}\right)$ and outputs the encoded message *W*. Upon observing local information $Y^{n}$ and receiving *W*, the decoder reconstructs the source sequences as $\left({\hat{X}}_{1}^{n},{\hat{X}}_{2}^{n}\right)$ drawn values from ${\hat{X}}_{1}\times {\hat{X}}_{2}$, within distortions $D_{1}$ and $D_{2}$, respectively. Given the reconstructions, the classifier aims to recover the semantic information as ${\hat{S}}^{n}$ from alphabet $\hat{S}$ with distortion constraint $D_{s}$. Here, for simplicity, we assume a perfect classifier; i.e., it is equivalent to recovering ${\hat{S}}^{n}$ directly at the decoder.

Formally, an $\left(n,2^{nR}\right)$ code is defined by the encoding function

$$\begin{array}{c} En:X_{1}^{n}\times X_{2}^{n}\times Y^{n}\to \left\{1,2,\cdots ,2^{nR}\right\} \end{array}$$

and the decoding function

$$\begin{array}{c} De:\left\{1,2,\cdots ,2^{nR}\right\}\times Y^{n}\to {\hat{X}}_{1}^{n}\times {\hat{X}}_{2}^{n}\times {\hat{S}}^{n}, \end{array}$$

where *R* is the coding rate. Let $R^{+}$ be the set of non-negative real numbers. We consider bounded per-letter distortion functions $d_{1}:X_{1}\times {\hat{X}}_{1}\to R^{+}$, $d_{2}:X_{2}\times {\hat{X}}_{2}\to R^{+}$, and $d_{s}:S\times \hat{S}\to R^{+}$. The distortions between two length-*n* sequences are defined as average distortion, e.g.,

$$\begin{array}{c} d_{1}\left(x_{1}^{n},{\hat{x}}_{1}^{n}\right)≜\frac{1}{n}\sum_{i=1}^{n}d_{1}\left(x_{1,i},{\hat{x}}_{1,i}\right). \end{array}$$

A non-negative rate–distortion tuple $\left(R,D_{1},D_{2},D_{s}\right)$ is said to be *achievable* if for sufficiently large *n*, there exists an $\left(n,2^{nR}\right)$ code such that

$$\begin{array}{c} \lim_{n\to \infty }Ed_{1}\left(X_{1}^{n},{\hat{X}}_{1}^{n}\right)\leq D_{1},\;\lim_{n\to \infty }Ed_{2}\left(X_{2}^{n},{\hat{X}}_{2}^{n}\right)\leq D_{2},\;\lim_{n\to \infty }Ed_{s}\left(S^{n},{\hat{S}}^{n}\right)\leq D_{s}. \end{array}$$

The semantic rate–distortion function $R(D_{1},D_{2},D_{s})$ is the infimum of coding rate *R* for distortions $\left(D_{1},D_{2},D_{s}\right)$ such that the quadruple $\left(R,D_{1},D_{2},D_{s}\right)$ is achievable. The goal of this paper is to completely characterize $R(D_{1},D_{2},D_{s})$.

### 2.2. Preliminaries

#### 2.2.1. Conditional Rate–Distortion Function

The elegant rate–distortion function was investigated and fully characterized in [2]. Assume the length-*n* source sequence $X^{n}$ is independent and identically distributed (i.i.d.) over X with generic random variable *X* and let $d:X\times \hat{X}\to R^{+}$ be a bounded per-letter distortion measure. The rate–distortion function for a given distortion criterion *D* is given by

(1) $$R\left(D\right)=\min_{p\left(\hat{x}|x\right):Ed\left(X,\hat{X}\right)\leq D}I\left(X;\hat{X}\right).$$

It was proved in [2] and also introduced in [22,23] that R(D) is a *non-increasing* and *convex* function of *D*.

If both the encoder and decoder are allowed to observe side information $Y^{n}$ (with generic variable *Y* over Y jointly distributed with *X*), then the tradeoff is called the conditional rate–distortion function [15,16], which is characterized as

(2) $$R_{X|Y}\left(D\right)=\min_{p\left(\hat{x}|x,y\right):Ed\left(X,\hat{X}\right)\leq D}I\left(X;\hat{X}|Y\right).$$

If (X,Y) is a *doubly symmetric binary source* (DSBS) with parameter $p_{0}$, i.e.,

(3) $$p\left(x,y\right)=\left\{\begin{array}{cc} \frac{1-p_{0}}{2}, & \;\mathrm{if}\;x=y\\ \frac{p_{0}}{2}, & \;\mathrm{if}\;x\neq y. \end{array}\right.$$

then the conditional rate–distortion function is given in [16] by

(4) $$R_{X|Y}\left(D\right)=\left[h_{b}\left(p_{0}\right)-h_{b}\left(D\right)\right]\cdot 1_{0\leq D\leq p_{0}},$$

where $h_{b}\left(q\right)=-q\log q-\left(1-q\right)\log\left(1-q\right)$ is the entropy for a Bernoulli(*q*) distribution and $1_{A}$ is the indicator function of whether event *A* happens.

#### 2.2.2. Rate–Distortion Function with Two Constraints

This scenario was discussed in [23] that one wishes to encode the i.i.d. source sequence $X^{n}$ at rate *R* and recover two reconstructions ${\hat{X}}_{a}^{n}$ and ${\hat{X}}_{b}^{n}$ with distortion criteria $Ed_{a}\left(X^{n},{\hat{X}}_{a}^{n}\right)\leq D_{a}$ and $Ed_{b}\left(X^{n},{\hat{X}}_{b}^{n}\right)\leq D_{b}$, respectively. The rate–distortion function is given by

(5) $$R_{2d}\left(D_{a},D_{b}\right)=\min_{\begin{array}{c} p({\hat{x}}_{a},{\hat{x}}_{b}|x):\;\\ Ed_{a}\left(X,{\hat{X}}_{a}\right)\leq D_{a}\\ Ed_{b}\left(X,{\hat{X}}_{b}\right)\leq D_{b} \end{array}}I\left(X;{\hat{X}}_{a},{\hat{X}}_{b}\right).$$

Comparing (1) and (5), one easily obtains that

$$\max\left\{R\left(D_{a}\right),R\left(D_{b}\right)\right\}\leq R_{2d}\left(D_{a},D_{b}\right)\leq R\left(D_{a}\right)+R\left(D_{b}\right).$$

For the special case where ${\hat{X}}_{a}={\hat{X}}_{b}$ and $d_{a}\left(x,\hat{x}\right)=d_{b}\left(x,\hat{x}\right)$ for all x∈X and $\hat{x}\in {\hat{X}}_{a}$, it suffices to recover only one sequence ${\hat{X}}_{a}^{n}={\hat{X}}_{b}^{n}$ with distortion $\min\{D_{a},D_{b}\}$. Then, both distortion constraints are satisfied since $Ed_{a}\left(X^{n},{\hat{X}}_{a}^{n}\right)=Ed_{b}\left(X^{n},{\hat{X}}_{b}^{n}\right)=\min\left\{D_{a},D_{b}\right\}$, which is upper bounded by both $D_{a}$ and $D_{b}$. This implies

(6) $$R_{2d}\left(D_{a},D_{b}\right)=R\left(\min\left\{D_{a},D_{b}\right\}\right)=\max\left\{R\left(D_{a}\right),R\left(D_{b}\right)\right\},$$

where the second equality follows from the non-increasing property of R(D).

When side information is available at the decoder for only one of the two reconstructions, e.g., ${\hat{X}}_{b}$, it was proved in [18] that successive encoding (first ${\hat{X}}_{a}$, then ${\hat{X}}_{b}$) is optimal. For the case where the two reconstructions have access to different side information, respectively, the rate–distortion tradeoff was characterized in [22].

#### 2.2.3. Rate–Distortion Function of Two Sources

The problem of compressing two i.i.d. source sequences $X_{a}^{n}$ and $X_{b}^{n}$ at the same encoder is considered in [23] [Problem. 10.14]. The rate–distortion function is given therein, i.e.,

(7) $$R_{2s}\left(D_{a},D_{b}\right)=\min_{\begin{array}{c} p({\hat{x}}_{a},{\hat{x}}_{b}|x_{a},x_{b}):\;\\ Ed_{a}\left(X_{a},{\hat{X}}_{a}\right)\leq D_{a}\\ Ed_{b}\left(X_{b},{\hat{X}}_{b}\right)\leq D_{b} \end{array}}I\left(X_{a},X_{b};{\hat{X}}_{a},{\hat{X}}_{b}\right).$$

It is also shown that for two independent sources, compressing simultaneously is the same as compressing separately in terms of the rate and distortions, i.e.,

(8) $$R_{2s}\left(D_{a},D_{b}\right)=R\left(D_{a}\right)+R\left(D_{b}\right).$$

If the two sources are dependent, the equality in (8) can be false, and the Slepian–Wolf rate region [19] indicates that joint entropy of the two source variables is sufficient and optimal for lossless reconstructions. Taking into account distortions, Gray showed via an example in [16] that the compression rate can be strictly larger than $R\left(D_{a}\right)+R_{X_{b}|X_{a}}\left(D_{b}\right)$ in general. At last, some related results for compressing compound sources can be found in [24].

## 3. Optimal Rate–Distortion Tradeoff

### 3.1. The Complete Rate–Distortion Function

**Theorem 1.**  *The complete rate–distortion function for semantic compression with side information is given as the solution to the following optimization problem*

(9) $$\begin{array}{ccc} R(D_{1},D_{2}, & D_{s}) & =\min I(X_{1},X_{2};{\hat{X}}_{1},{\hat{X}}_{2},\hat{S}|Y) \end{array}$$

$$\begin{array}{ccc} & s.t. & Ed_{1}\left(X_{1},{\hat{X}}_{1}\right)\leq D_{1},\\ & & \;Ed_{2}\left(X_{2},{\hat{X}}_{2}\right)\leq D_{2},\\ & & \;Ed_{s}^{\prime }\left(X_{1},\hat{S}\right)\leq D_{s}, \end{array}$$

*where the minimum is taken over all conditional pmf $p({\hat{x}}_{1},{\hat{x}}_{2},\hat{s}|x_{1},x_{2},y)$ and the modified distortion measure is defined by*

(10) $$d_{s}^{\prime }\left(x_{1},\hat{s}\right)=\frac{1}{p(x_{1})}\sum_{s\in S}p\left(x_{1},s\right)d_{s}\left(s,\hat{s}\right).$$

**Proof.**  We provide a rigorous technical proof in Appendix A. □

**Remark 1.**  *We can interpret the problem as the combination of rate–distortion with two sources ($X_{1}$ and $X_{2}$), rate–distortion with two constraints ($X_{1}$ is recovered with two constraints $D_{1}$ and $D_{s}$), and conditional rate–distortion (conditioning on Y). Then, Theorem 1 can be obtained informally by combining the rate–distortion functions in* (2)*, *(5)*, and *(7)*.*

**Remark 2.**  *Theorem 1 considers the observation of two semantic segments, and it can be easily extended to arbitrary T segments by expanding the notations $X_{t}$ and ${\hat{X}}_{t}$, 1≤t≤T.*

### 3.2. Some Properties

Similar to the rate–distortion function (1), we collect some properties in the following lemma. The proof simply follows the same procedure as that for (1) in [2,22,23]. We omit the details here.

**Lemma 1.**  *The rate–distortion function $R(D_{1},D_{2},D_{s})$ is non-increasing and convex in $\left(D_{1},D_{2},D_{s}\right)$.*

Recall from (8) that compressing two independent sources is the same as compressing them simultaneously. Then, one may naturally ask whether the optimality of separate compression remains to hold here. We answer the question in the following lemma.

**Lemma 2.**  *If $X_{1}-Y-X_{2}$ forms a Markov chain, then*

$$R\left(D_{1},D_{2},D_{s}\right)=R_{2d,X_{1}|Y}\left(D_{1},D_{s}\right)+R_{X_{2}|Y}\left(D_{2}\right),$$

*where the conditional rate–distortion function with two constraints is given by*

$$R_{2d,X_{1}|Y}\left(D_{1},D_{s}\right)=\min_{\begin{array}{c} p({\hat{x}}_{1},\hat{s}|x_{1},y):\\ Ed_{1}\left(X_{1},{\hat{X}}_{1}\right)\leq D_{1}\\ Ed_{s}^{\prime }\left(X_{1},\hat{S}\right)\leq D_{s} \end{array}}I\left(X_{1};{\hat{X}}_{1},\hat{S}|Y\right)$$

*and the conditional rate–distortion function is given in* (2) *and can be written as*

$$R_{X_{2}|Y}\left(D\right)=\min_{p\left({\hat{x}}_{2}|x_{2},y\right):Ed_{2}\left(X_{2},{\hat{X}}_{2}\right)\leq D_{2}}I\left(X_{2};{\hat{X}}_{2}|Y\right).$$

**Proof.**  The proof is given in Appendix B. □

**Remark 3.**  *Compared to the unconditional independence assumption required for the equality in* (8)*, the optimality of separate compression in Lemma 2 requires conditional independence $I(X_{1};X_{2}|Y)=0$, which is equivalent to the Markov chain $X_{1}-Y-X_{2}$. Moreover, this is the necessary and sufficient condition for separate compression optimality in the presence of side information Y. This is intuitive because both the encoder and decoder have access to the side information Y, meaning the rate–distortion tradeoff is evaluated under the conditional probability space given Y.*

*Direct (unconditional) independence of $X_{1}$ and $X_{2}$ is neither necessary nor sufficient for separate compression to be optimal here. On one hand, if $X_{1}$ and $X_{2}$ are unconditionally independent but conditionally dependent given Y (for instance, when $X_{1}$ and $X_{2}$ are independent Bernoulli (0.5) variables and $Y=X_{1}⊕X_{2}$), joint compression conditioned on Y can exploit this conditional correlation to achieve a strictly lower rate than separate compression. On the other hand, $X_{1}$ and $X_{2}$ can be unconditionally dependent, but as long as their mutual correlation is fully mediated by the side information Y (i.e., $X_{1}-Y-X_{2}$ holds), separate compression remains optimal.*

### 3.3. Rate–Distortion Function for Semantic Information Only

The indirect rate–distortion problem can be viewed as a special case of Theorem 1 that only recovers the semantic information *S*; i.e., $X_{2}$ and *Y* are constants and $D_{1}=\infty$. Denote the minimum achievable rate for a given distortion constraint $D_{s}$ by $R_{s}\left(D_{s}\right)$.

Consider doubly symmetric binary sources *S* and $X_{1}$, i.e.,

(11) $$\begin{array}{c} p\left(s,x_{1}\right)=\left\{\begin{array}{cc} \frac{1-p}{2}, & \;\mathrm{if}\;s=x_{1}\\ \frac{p}{2}, & \;\mathrm{if}\;s\neq x_{1}. \end{array}\right. \end{array}$$

Without loss of generality, assume p≤0.5, which means that $X_{1}$ has a higher probability to reflect the same value as *S*. Let $d_{s}:S\times \hat{S}\to \left\{0,1\right\}$ be the Hamming distortion measure. Then, the evaluation of $R_{s}\left(D_{s}\right)$ is given in the following lemma.

Let $R_{d_{s}^{\prime }}\left(\cdot \right)$ be the Shannon rate–distortion function in (1) under the distortion measure $d_{s}^{\prime }$ (c.f. (10)). For notational simplicity, and for $D_{s}\geq p$, define

(12) $$D_{s}^{0}≜\frac{D_{s}-p}{1-2p}.$$

**Lemma 3.**  *For binary sources in* (11) *and Hamming distortion, the rate–distortion function for semantic information is*

(13) $$R_{s}\left(D_{s}\right)=R_{d_{s}^{\prime }}\left(D_{s}\right),$$

*where $R_{d_{s}^{\prime }}\left(D_{s}\right)=R\left(D_{s}^{0}\right)=\left[1-h_{b}\left(\frac{D_{s}-p}{1-2p}\right)\right]\cdot 1_{p\leq D_{s}\leq 0.5}.$*

**Proof.**  The evaluation of $R_{s}\left(D_{s}\right)$ was given in [25]. A simpler proof is in Appendix C. □

**Remark 4.**  *By the properties of the rate–distortion function in* (1) *and the linearity between $D_{s}^{0}$ and $D_{s}$, we see that $R_{s}\left(D_{s}\right)$ is also non-increasing and convex in $D_{s}$.*

**Remark 5.**  *It is easy to check that $\frac{D-p}{1-2p}<D$ for D<0.5. This implies that $R_{s}\left(D\right)>R\left(D\right)$ for D<0.5, where R(D) is the Shannon rate–distortion function* (1)*. The inequality is intuitive from the data processing inequality that under the same distortion constraint D, recovering S directly (with rate R(D)) is easier than recovering it from the observation $X_{1}$ (with rate $R_{s}\left(D\right)$). Moreover, we see from the lemma that $D_{s}\geq p$, which means that the semantic information can never be losslessly recovered for p>0. This can be deduced from the fact that even though we know the complete information of $X_{1}$, the best distortion for reconstructing S is the distortion between S and $X_{1}$, which is equal to p.*

**Figure 2:**
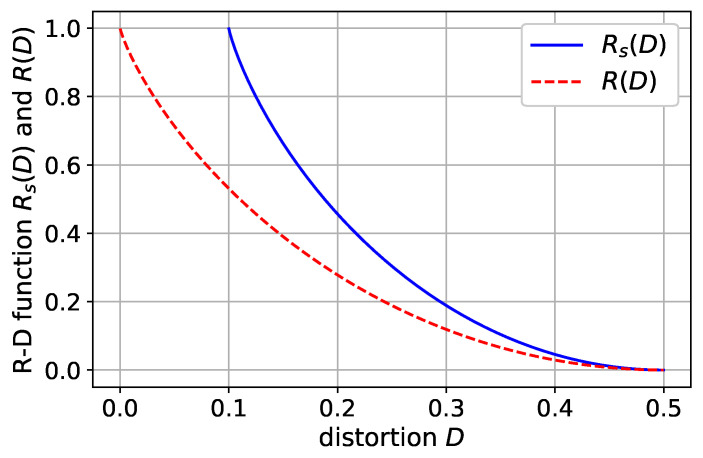
Comparison of rate–distortion functions $R_{s}\left(D\right)$ and R(D) for p=0.1.

*The rate–distortion functions $R_{s}\left(D\right)$ and R(D) are illustrated in Figure 2 for p=0.1, which verifies the above observations. For general source and distortion measure, we have $R_{s}\left(D\right)\geq R\left(D\right)$ where the equality holds only when $X_{1}$ determines S. This can be easily proved by the data processing inequality, and we omit the details here.*

**Remark 6.**  *We can imagine that $d_{s}^{\prime }$ measures the distortion between the observation and reconstruction of semantic information. Furthermore, it was shown in [10,11] that $d_{s}$ and $d_{s}^{\prime }$ measure equivalent distortions, i.e.,*

$$Ed_{s}^{\prime }\left(X_{1},\hat{S}\right)=Ed_{s}\left(S,\hat{S}\right),\;\;\;\;Ed_{s}^{\prime }\left(X_{1}^{n},{\hat{S}}^{n}\right)=Ed_{s}\left(S^{n},{\hat{S}}^{n}\right).$$

*Then, we can regard the system of compressing $X_{1}^{n}$ and reconstructing ${\hat{S}}^{n}$ as the Shannon rate–distortion problem with distortion measure $d_{s}^{\prime }$. Thus, $R_{s}\left(D_{s}\right)$ is equivalent to the Shannon rate–distortion function in* (1) *under distortion measure $d_{s}^{\prime }$, which rigorously proves* (13)*.*

## 4. Case Studies

### 4.1. Binary Semantic Sources

Assume *S* and $X_{1}$ are doubly symmetric binary sources with distribution in (11) and $X_{2}$ and *Y* are both Bernoulli $\left(\frac{1}{2}\right)$ sources. The reconstructions are all binary, i.e., ${\hat{X}}_{1}={\hat{X}}_{2}=\hat{S}=\left\{0,1\right\}$. The distortion measures $d_{1},d_{2}$, and $d_{s}$ are all assumed to be Hamming distortion. We further assume that any two of $X_{1}$, $X_{2}$, and *Y* are doubly symmetric binary distributed with parameters $p_{1},p_{2}$, and $p_{3}$, respectively. Specifically, $\left(X_{1},X_{2}\right)∼\mathrm{DSBS}\left(p_{1}\right)$, $\left(X_{1},Y\right)∼\mathrm{DSBS}\left(p_{2}\right)$, $\left(X_{2},Y\right)∼\mathrm{DSBS}\left(p_{3}\right)$.

#### 4.1.1. Conditionally Independent Binary Sources

We further assume the Markov chain $X_{1}-Y-X_{2}$; i.e., $X_{1}$ and $X_{2}$ are conditionally independent given *Y*. This assumption coincides with the intuitive understanding of $X_{1}$ and $X_{2}$ in Section 2.1, indicating that the most relevant semantic segment can be independent with the remaining parts. (Note that the Markov chain $X_{1}-Y-X_{2}$ indicates $p_{1}=p_{2}★p_{3}≜p_{2}\left(1-p_{3}\right)+p_{3}\left(1-p_{2}\right)$.) Then, from Lemma 2, compressing $X_{1}^{n}$ and $X_{2}^{n}$ simultaneously is the same as compressing them separately in terms of the optimal compression rate and distortions, which implies the following theorem.

**Theorem 2.**  *For the conditionally independent sources satisfying the Markov chain $X_{1}-Y-X_{2}$, the rate–distortion function is given by*

$$\begin{array}{c} R\left(D_{1},D_{2},D_{s}\right)=\left[h_{b}\left(p_{3}\right)-h_{b}\left(D_{2}\right)\right]\cdot 1_{0\leq D_{2}\leq p_{3}}+\left[h_{b}\left(p_{2}\right)-h_{b}\left(\min\{D_{1},D_{s}^{0}\}\right)\right]\cdot 1_{0\leq \min\left\{D_{1},D_{s}^{0}\right\}\leq p_{2}}, \end{array}$$

*where $D_{s}^{0}=\frac{D_{s}-p}{1-2p}$ is defined in* (12)*.*

**Proof.**  The rate–distortion function in Theorem 1 satisfies

$$\begin{array}{cc} R(D_{1},D_{2},D_{s})\; & =R_{2d,X_{1}|Y}\left(D_{1},D_{s}\right)+R_{X_{2}|Y}\left(D_{2}\right)\\ & =\min_{\begin{array}{c} Ed_{1}\left(X_{1},{\hat{X}}_{1}\right)\leq D_{1}\\ Ed_{s}^{\prime }\left(X_{1},\hat{S}\right)\leq D_{s} \end{array}}I\left(X_{1};{\hat{X}}_{1},\hat{S}|Y\right)+\min_{Ed_{2}\left(X_{2},{\hat{X}}_{2}\right)\leq D_{2}}I\left(X_{2};{\hat{X}}_{2}|Y\right)\\ & =\left[h_{b}\left(p_{2}\right)-h_{b}\left(\min\{D_{1},D_{s}^{0}\}\right)\right]\cdot 1_{0\leq \min\left\{D_{1},D_{s}^{0}\right\}\leq p_{2}}\\ & \;\;\;\;\;+\left[h_{b}\left(p_{3}\right)-h_{b}\left(D_{2}\right)\right]\cdot 1_{0\leq D_{2}\leq p_{3}}, \end{array}$$

where the last step follows from the rate–distortion functions in (4), (6) and (13). □

#### 4.1.2. Binary Classification of Integers

We consider the classification of integers as even or odd. Let $X_{1}$ be uniformly distributed over $X_{1}=\left[1:N\right]$ with N≥4 being an even integer. The semantic information *S* is a binary random variable that probabilistically indicates whether $X_{1}$ is even or odd. The transition probability can be defined by a parameter *p*, which is similar to that in (11) by replacing the value of $X_{1}$ with “even” and “odd”. The binary side information *Y* is correlated with $X_{1}$ and also indicates its oddity (even/odd) with parameter $p_{2}$.

Assume the Markov chain $X_{1}-Y-X_{2}$ holds, and the Bernoulli($\frac{1}{2}$) source $X_{2}$ is independent with *Y*. We can verify that $X_{2}$ is independent with $\left(X_{1},Y\right)$. By Lemma 2, compressing $X_{1}^{n}$ and $X_{2}^{n}$ simultaneously is the same as compressing them separately. For simplicity, we consider only small distortions such that $\left(D_{1},D_{2},D_{s}\right)\in D_{1}$ where

(14) $$\begin{array}{cc} D_{1} & =\left\{\left(D_{1},D_{2},D_{s}\right):0\leq \min\left\{D_{1},D_{s}^{0}\right\}\leq \frac{2\left(N-1\right)p_{2}}{N}\;\mathrm{and}\;0\leq D_{2}\leq 0.5\right\}. \end{array}$$

**Theorem 3.**  *For $\left(D_{1},D_{2},D_{s}\right)\in D_{1}$, the rate–distortion function for integer classification is*

$$\begin{array}{c} R\left(D_{1},D_{2},D_{s}\right)=\left[h_{b}\left(p_{2}\right)+\log\left(N/2\right)-h_{b}\left(\min\left\{D_{1},D_{s}^{0}\right\}\right)-D_{1}\log\left(N-1\right)\right]+\left[1-h_{b}\left(D_{2}\right)\right], \end{array}$$

*where $D_{s}^{0}=\frac{D_{s}-p}{1-2p}$ is defined in* (12)*.*

**Proof.**  The proof is given in Appendix D. □

#### 4.1.3. Numerical Results for Binary Classification

**Figure 3:**
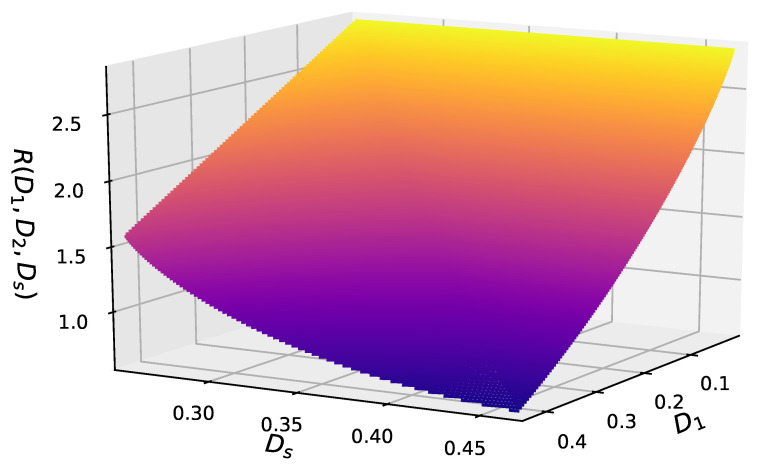
The rate–distortion function for integer classification with $p=p_{1}=p_{2}=0.25$, $D_{2}=0.5$, and N=8.

The rate–distortion function for integer classification in Theorem 3 with $p=p_{1}=p_{2}=0.25$, $D_{2}=0.5$, and N=8 is illustrated in Figure 3. Note that in Theorem 3, $D_{2}=0.5$ indicates that $X_{2}$ can be recovered by random guessing, which further implies that $X_{2}$ can also be regarded as side information at both sides.

By comparing the rates along the $D_{1}$ and $D_{s}$ axes in Figure 3, it is evident that recovering only the semantic information can reduce the coding rate compared to recovering the original source message.

### 4.2. Gaussian Sources

Assume *S* and $X_{1}$ are jointly Gaussian sources with zero mean and covariance matrix

(15) $$\left[\begin{array}{cc} \sigma_{S} & \sigma_{SX_{1}}\\ \sigma_{SX_{1}} & \sigma_{X_{1}} \end{array}\right].$$

Similarly, we assume the Markov chain $X_{1}-Y-X_{2}$ where $X_{2}$ and *Y* are jointly Gaussian sources with zero mean and covariance matrix

(16) $$\left[\begin{array}{cc} \sigma_{X_{2}} & \sigma_{X_{2}Y}\\ \sigma_{X_{2}Y} & \sigma_{Y} \end{array}\right].$$

Thus, $X_{1}$ is conditionally independent of $X_{2}$ given *Y*. Let the covariance of $X_{1}$ and *Y* be $\sigma_{X_{1}Y}$. The reconstructions are real scalars, i.e., ${\hat{X}}_{1}={\hat{X}}_{2}=\hat{S}=R$, and the distortion metrics are squared error.

#### 4.2.1. Theoretical Bounds and Dominance Analysis

Given the Markov chain, we see from Lemma 2 that compressing $X_{1}^{n}$ and $X_{2}^{n}$ simultaneously is the same as compressing them separately in terms of the optimal compression rate and distortions. Then, we have the following theorem.

**Theorem 4.**  *For the Gaussian sources, if the Markov chain $X_{1}-Y-X_{2}$ holds, the rate–distortion function is*

(17) $$\begin{array}{c} R\left(D_{1},D_{2},D_{s}\right)=\frac{1}{2}{\left(\log\frac{\sigma_{X_{2}}-\frac{\sigma_{X_{2}Y}^{2}}{\sigma_{Y}}}{D_{2}}\right)}^{+}+\frac{1}{2}{\left[\log\max\left(\frac{\sigma_{X_{1}}-\frac{\sigma_{X_{1}Y}^{2}}{\sigma_{Y}}}{D_{1}},\frac{\sigma_{SX_{1}}^{2}\left(\sigma_{X_{1}}-\frac{\sigma_{X_{1}Y}^{2}}{\sigma_{Y}}\right)}{\sigma_{X_{1}}^{2}\left(D_{s}-D_{\mathrm{MMSE}}\right)}\right)\right]}^{+}, \end{array}$$

*where ${\left(x\right)}^{+}=\max\left(x,0\right)$ and $D_{\mathrm{MMSE}}$ is the minimum mean squared error for estimating S from $X_{1}$, which is given by*

(18) $$D_{\mathrm{MMSE}}=\sigma_{S}-\frac{\sigma_{SX_{1}}^{2}}{\sigma_{X_{1}}}.$$

**Proof.**  The proof is given in Appendix E. □

To theoretically characterize the dominant roles of $D_{1}$ and $D_{s}$ in Theorem 4, we compare the two terms inside the max(·,·) operator in (17). The boundary between the reconstruction-dominant and semantic-dominant regions is given by the linear threshold:

(19) $$D_{s}=D_{MMSE}+D_{1}\frac{\sigma_{SX_{1}}^{2}}{\sigma_{X_{1}}^{2}}.$$

Specifically, when $D_{s}\geq D_{MMSE}+D_{1}\frac{\sigma_{SX_{1}}^{2}}{\sigma_{X_{1}}^{2}}$, the reconstruction constraint $D_{1}$ dominates the rate; otherwise, the semantic constraint $D_{s}$ dominates the rate.

#### 4.2.2. Numerical Results for Gaussian Sources

**Figure 4:**
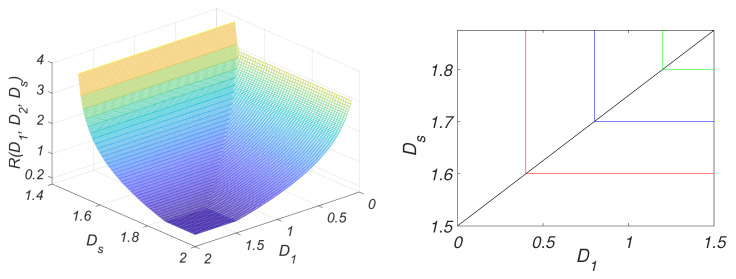
The rate–distortion function for Gaussian sources and its contour plot, for $\sigma_{S}=\sigma_{X_{1}}=\sigma_{X_{2}}=\sigma_{Y}=1$, $\sigma_{SX_{1}}=\sigma_{X_{1}Y}=\sigma_{X_{2}Y}=2$, and $D_{2}=1$.

We then present the numerical analysis of the Gaussian rate–distortion function in Theorem 4, where we take all of the variances as 2, all of the covariances as 1, and $D_{2}=1$. The resultant optimal tradeoff between the coding rate $R(D_{1},D_{2},D_{s})$ and distortions $\left(D_{1},D_{s}\right)$ is illustrated in Figure 4. It can be observed that the rate–distortion function is decreasing and convex in $\left(D_{1},D_{s}\right)$, and the minimum rate is given as $R_{X_{2}|Y}\left(D_{2}\right)=\frac{1}{2}{\left(\log\frac{\sigma_{X_{2}}-\frac{\sigma_{X_{2}Y}^{2}}{\sigma_{Y}}}{D_{2}}\right)}^{+}=0.20.$

The corresponding contour plot is also shown in Figure 4, where the slanted line represents the theoretical boundary in (19). Under our specific numerical setup, this boundary simplifies to the linear relation $D_{s}=1.5+0.25D_{1}$, which perfectly matches the slanted line starting at $D_{s}=1.5$ when $D_{1}=0$ and passing through the corner points of the L-shaped contour lines. As predicted by our theoretical analysis, when the distortion pair $\left(D_{1},D_{s}\right)$ lies in the reconstruction-dominant region above this slanted line (where $D_{s}\geq 1.5+0.25D_{1}$), the rate is determined solely by the reconstruction distortion constraint of $X_{1}$. Conversely, in the semantic-dominant region below the slanted line (where $D_{s}<1.5+0.25D_{1}$), the rate only needs to meet the distortion constraint $D_{s}$ for reconstructing the semantic information *S*.

## 5. Experimental Results and Discussion

This section validates the theoretical analysis by implementing a deep learning-based image compression scheme for a classification task.

### 5.1. Deep Learning-Based Classification-Oriented Image Compression Scheme

**Figure 5:**
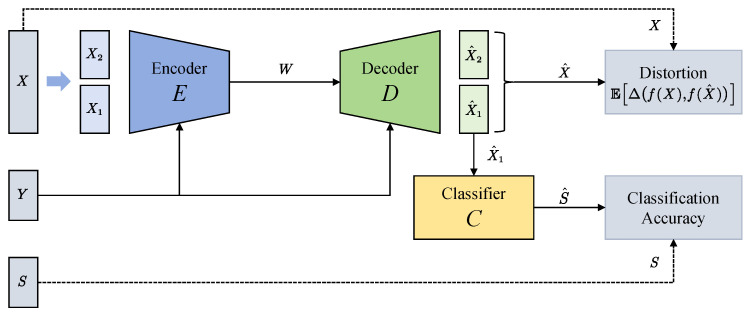
Illustration of rate–distortion classification setup.

By employing an autoencoder (AE), the lossy compression procedure can be realized through a parameterized encoder and decoder, which is denoted as $p_{\hat{X}|XY}\left(\hat{X}|X,Y\right)$. Let $\phi =\{\phi_{e},\phi_{d}\}$ represent the parameters of the entire encoder–decoder network. Similar to [13,26], we adopt a neural network with a stochastic encoder and decoder together with universal quantization [27]. As shown in Figure 5, the system consists of an encoder *E*, a decoder *D*, and a classifier *C*.

In the experiments, we take the central region of the image as the most relevant semantic segment, i.e., $X_{1}$, and the surrounding region of the image is regarded as $X_{2}$. The size of $X_{1}$ is one half of each image; e.g., for an image of size 32×32, the central region of 16×16 is designated as $X_{1}$. Both $X_{1}$ and $X_{2}$ are fed into two identical encoder–decoder pairs without parameter sharing. In addition, the top 1/3 of each image is taken as side information *Y*, which is accessible by both the encoder and the decoder. The classifier takes only the estimation ${\hat{X}}_{1}$ of the most relevant semantic region as its input and predicts a vector $\hat{S}$, where each entry represents the probability that the image belongs to each class. The true label *S* and the predicted label $\hat{S}$ are used to compute the classification accuracy. Moreover, we take the conventional mean squared error (MSE) as the distortion measure, i.e., $E\left(∥X-\hat{X}{∥}^{2}\right)$. As for the classification evaluation, we employ the commonly used cross-entropy (CE) loss, i.e.,

(20) $$\begin{array}{c} \mathrm{CE}\left(S,\hat{S}\right)=\sum_{S}\sum_{{\hat{X}}_{1}}p_{\phi }\left(S,\hat{X}\right)\log\frac{1}{p_{\psi }\left(S|{\hat{X}}_{1}\right)}=-\frac{1}{N}\sum_{{\hat{X}}_{1}}\log p_{\psi }\left(i_{X_{1}}|{\hat{X}}_{1}\right), \end{array}$$

where $\hat{S}$ denotes the output vector of the classifier given ${\hat{X}}_{1}$, and its *i*-th entry corresponds to the conditional probability of the *i*-th class, i.e., $p_{\psi }\left(S|{\hat{X}}_{1}\right)$, with ψ being the parameters of the classifier. Given encoder–decoder parameter pair $\left(\phi_{e},\phi_{d}\right)$, the reconstruction ${\hat{X}}_{1}=D_{\phi_{d}}\left(E_{\phi_{e}}\left(X_{1}\right)\right)$ is a function of the corresponding input $X_{1}$. Let the label of $X_{1}$ be $i_{X_{1}}$, and let the batch size be *N*. If $S=i_{X_{1}}$, then $p_{\phi }\left(S,{\hat{X}}_{1}\right)=\frac{1}{N}$; otherwise, $p_{\phi }\left(S,{\hat{X}}_{1}\right)=0$.

To evaluate the compression rate *R*, we follow the settings in [13,26] and define *R* as the upper bound $k\times \log_{2}\left(L\right)$, where *k* denotes the dimensionality of the encoder output and *L* is the number of quantization levels for each entry. As discussed in [28], setting *R* to its upper bound greatly simplifies the framework and is found to be only slightly suboptimal. Finally, given a rate $R=k\times \log_{2}\left(L\right)$, the overall loss function L of the deep learning-based image compression framework is defined as a weighted sum of MSE and CE, i.e.,

(21) $$\begin{array}{c} L≜\lambda_{d}\;E\left(∥X-\hat{X}∥\right)+\lambda_{c}\;\mathrm{CE}\left(S,\hat{S}\right), \end{array}$$

where $\lambda_{d}$ and $\lambda_{c}$ are hyper-parameters balancing the weights of distortion and classification losses, respectively.

### 5.2. Results and Discussion

**Figure 6:**
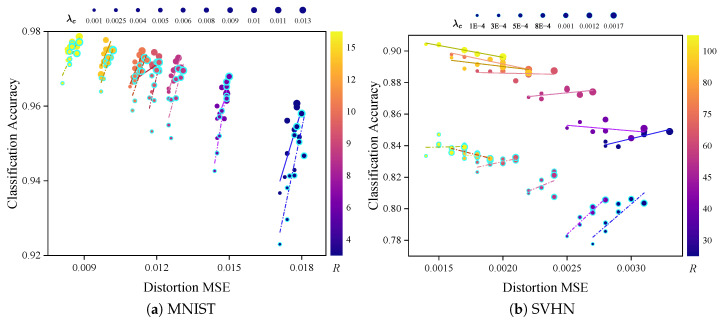
The classification accuracy versus distortion with varying rates *R* and weights $\lambda_{c}$. The results of Case A and Case B are denoted by points with and without black outline, respectively. The fitting curve is also plotted for each *R*, where the solid and dashed lines represent, respectively Case B and Case A.

Figure 6 illustrates the tradeoff between MSE and classification accuracy on the MNIST and SVHN datasets, where $\lambda_{d}$ is fixed to 1 and the variations are introduced by changing $\lambda_{c}$ and *R*. Note that each color represents a fixed compression rate *R*, which is achieved by applying the same quantizer throughout the experiments. For the MNIST and SVHN datasets, we take ${\left(k,L\right)}_{R}\in \left\{{\left(2,4\right)}_{4},{\left(3,4\right)}_{6},{\left(4,4\right)}_{8},{\left(5,4\right)}_{10},{\left(3,8\right)}_{9},{\left(4,8\right)}_{12},{\left(5,8\right)}_{15}\right\}$, respectively, ${\left(k,L\right)}_{R}{\in \{\left(10,8\right)}_{30},{\left(15,8\right)}_{45},{\left(20,8\right)}_{60},{\left(25,8\right)}_{75},{\left(10,16\right)}_{40},{\left(20,16\right)}_{80},{\left(25,16\right)}_{100}\}$. Moreover, the weights $\lambda_{c}\in \{0.001,0.0025,0.004,0.005,0.006,0.008,0.009,0.01,0.011,0.013\}$ and $\lambda_{c}\in \{0.0001,0.0003,0.0005,0.0008,0.001,0.0012,0.0017\}$ are utilized for the MNIST and SVHN datasets, respectively. As shown in Figure 6, each point corresponds to an encoder–decoder pair and a classifier jointly trained with given *R* and $\lambda_{c}$. For fair comparison, consider the following two cases:

- **Case A:** the classifier takes only ${\hat{X}}_{1}$ as input;
- **Case B:** the classifier takes the pair $({\hat{X}}_{1}$, ${\hat{X}}_{2})$ as input.

The results of Case A are notably marked with a black outline. Note that the classification accuracy corresponds to $D_{s}$, while the MSE distortions for Cases A and B correspond to $D_{1}$ and $D_{2}$ in Theorem 1, respectively. Next, we discuss the experimental results in detail.

#### 5.2.1. Positive Impact of Side Information

**Figure 7:**
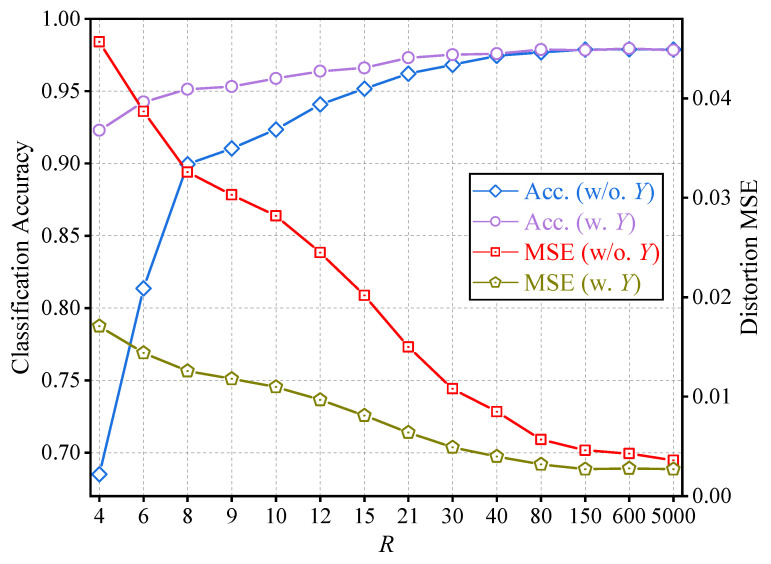
Illustration of performance improvement with side information on the MNIST dataset, when R=4, $\lambda_{d}=1$ and $\lambda_{c}=0.001$.

Figure 7 compares the distortion MSE and classification accuracy of the semantic compression scheme when side information is incorporated versus when it is excluded. As expected, incorporating side information leads to improvements in both distortion MSE and classification accuracy, and these gains are more substantial in the low-rate regime. The performance gap between the two settings gradually diminishes with growing rate *R*. This indicates that for sufficiently large rates, both classification and distortion have already reached satisfactory levels, thereby reducing the relative benefit provided by side information.

#### 5.2.2. Semantic Relevance Preserves Classification Accuracy

Recall that Case A employs only the most semantically relevant region, ${\hat{X}}_{1}$, as input to the classifier. As shown in Table 1, the distortion MSE of Case A varies within a narrow range compared to Case B. Although a decrease in classification accuracy is observed, it is generally limited to less than 7.5%. Specifically, Case A yields a modest average increase of 0.29% in distortion MSE compared to Case B, which is accompanied by a slight decrease in classification accuracy of 1.28% for the MNIST dataset. Moreover, for the SVHN dataset, Case A leads to an average reduction of 2.53% in MSE and an accuracy decline of 6.18%.

**Figure 8:**
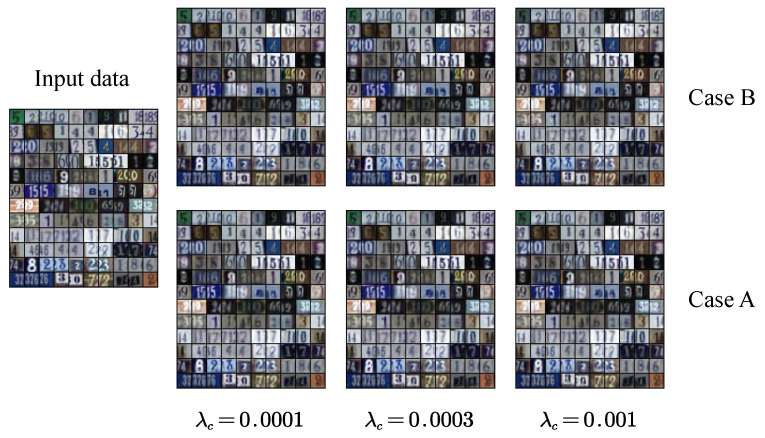
Visual reconstructions for the SVHN (R=30) dataset with different $\lambda_{c}$.

These results align with our theoretical analysis, confirming that retaining the most critical semantic features does not substantially compromise classification performance. In addition, the visualization results presented in Figure 8 further affirm our findings: across different values of $\lambda_{c}$, the reconstruction quality remains nearly identical between Case A and Case B.

#### 5.2.3. Trade-Off Between Classification Accuracy and Distortion

It can be observed from Figure 6 that for both Case A and Case B, higher classification accuracy is generally achieved at the expense of increased distortion MSE. As shown in Table 1, when $\lambda_{c}$ increases from 0.001 to 0.01 for the MNIST dataset, the MSE increases by 6.47% (Case A) and 4.09% (Case B), while the classification accuracy improves by 11.93% (Case A) and 4.74% (Case B). Moreover, it is noted that with rising *R*, the fitting curves shift upward and to the left corner, indicating that both distortion and classification accuracy improve with larger rates. Taking the MNIST dataset with $\lambda_{c}=0.001$ as an example, when rate *R* increases from 4 to 15, the MSE improves by 52.63% (Case A) and 51.46% (Case B), while the classification accuracy grows by 3.80% (Case A) and 4.67% (Case B). Similar trends can also be found for the SVHN dataset.

#### 5.2.4. The Role of Rate in Balancing Distortion and Classification

In addition to the MSE–accuracy pairs, Figure 6 also plots the corresponding MSE–accuracy fitting curves for each rate *R* with solid and dashed lines representing Case B and Case A, respectively.

We first discuss the results from the SVHN dataset (see Figure 6). Since the available transmission rate is limited in the low-rate regime (blue region), the compression system has to trade off between reconstruction quality (a smaller MSE corresponds to better reconstruction quality) and classification accuracy. In other words, relaxing the constraint on distortion (i.e., allowing a higher MSE) can contribute to improved classification accuracy. However, this tradeoff gradually weakens with increasing compression rate *R*. Notably, when *R* is sufficiently large (yellow region), the system would have adequate transmission resources, making it unnecessary to sacrifice reconstruction quality for accuracy improvement. Under such conditions, distortion MSE and classification accuracy exhibit a positive correlation; i.e., better reconstruction quality (lower MSE) results in higher classification accuracy (see the fitting curves with R>45).

In contrast, due to the considerably lower image resolution and dimensionality of the MNIST dataset (approximately one fourth that of SVHN dataset), even when the compression rate is relatively low, the system still has sufficient resources to optimize simultaneously the reconstruction quality and classification performance.

Moreover, the simple and regular structure of MNIST images renders the classifier relatively less sensitive to minor distortions, whereas the more diverse content in SVHN images leads to the classification accuracy having greater susceptibility to compression-induced loss. These inherent differences account for the distinct MSE–accuracy relationships observed in Figure 6.

#### 5.2.5. Practical Design Implications

The theoretical limits and case studies developed in this paper offer several critical guidelines for designing practical task-oriented semantic compression systems.

(i) Distributed Network Architecture: Lemma 2 suggests that if different semantic segments of an observation (e.g., foreground $X_{1}$ and background $X_{2}$ in video frames) are conditionally independent given temporal side information *Y* (e.g., key frames), we can safely design separate, parallel network branches to compress them without losing rate–distortion optimality.
(ii) Dynamic Bit Allocation: The dominance threshold boundary discussed in Theorem 4 indicates that when downstream task requirements are loose ($D_{s}$ is large), the compression network can safely optimize for reconstruction quality ($D_{1}$) using MSE loss alone. Conversely, when strict task accuracy is required, semantic classification loss must be heavily weighted to prevent task failure.
(iii) Side Information Utilization: The significant rate savings shown in Figure 7 demonstrate that predicting and sharing correlated side information *Y* at both ends is highly effective for both saving bandwidth and preserving task-classification accuracy.

## 6. Conclusions

This paper investigated the semantic rate–distortion theory with side information and the observation of two semantic variables. The corresponding optimal rate–distortion function was fully characterized, which particularly advanced the information-theoretic analysis of existing point-to-point semantic compression. In addition, we explicitly provided rate–distortion functions for binary and Gaussian sources under common distortion measures, and we revealed that the recovery of the semantic information is more efficient than reconstructing the original source. Moreover, experimental results on a classification-oriented image compression model validate the positive impact of side information, as anticipated by the theoretical analysis. For future work, our approach will be extended to more complex multi-user systems like the distributed compression system, the multiple description system, where the side information is available only at the decoder, etc.

## Figures and Tables

**Table 1:** Performance comparison of Case A and Case B for MNIST (R=4) and SVHN (R=30) datasets. Here, $\lambda_{d}$ is fixed to 1 and $\lambda_{c}$ varies. Moreover, ‘diff.’ denotes the relative difference between two cases, i.e., $\mathrm{diff}.=\frac{\mathrm{result}(\mathrm{Case}\;A)-\mathrm{result}(\mathrm{Case}\;B)}{\mathrm{result}(\mathrm{Case}\;B)}\times 100\%$.

	$\lambda_{c}$	Case	MSE	Accuracy	MSE diff.	Accuracy diff.
**(a) MNIST**	0.0010	Case B	0.0171	0.9162	0.00%	−7.40%
		Case A	0.0171	0.8484		
	0.0025	Case B	0.0173	0.9410	0.58%	−1.21%
		Case A	0.0174	0.9296		
	0.0060	Case B	0.0179	0.9517	−1.12%	−1.08%
		Case A	0.0177	0.9414		
	0.0100	Case B	0.0178	0.9597	1.69%	−1.35%
		Case A	0.0181	0.9467		
**(b) SVHN**	0.0010	Case B	0.0028	0.8397	−3.57%	−7.40%
		Case A	0.0027	0.7776		
	0.0030	Case B	0.0028	0.8425	0.00%	−6.75%
		Case A	0.0028	0.7856		
	0.0080	Case B	0.0030	0.8446	−3.33%	−5.52%
		Case A	0.0029	0.7980		
	0.0120	Case B	0.0031	0.8489	−3.23%	−5.04%
		Case A	0.0030	0.8061		

**Figure A1:**
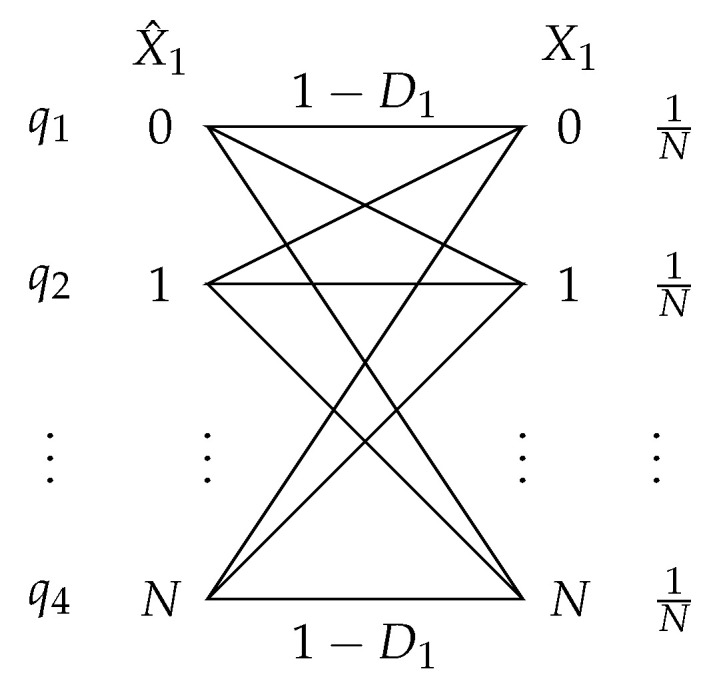
Test channel from ${\hat{X}}_{1}$ to $X_{1}$: $X_{1}∼$ Uniform($\frac{1}{N}$) and the transition probability $p(x_{1}|{\hat{x}}_{1})$ is given by (A11).

## Data Availability

No new data were created or analyzed in this study.

## Appendix A. Proof of Theorem 1

**Proof.** The achievability part is a straightforward extension of the joint typicality coding scheme for lossy source coding. We simply present the coding ideas and analysis as follows. Fix the conditional pmf $p({\hat{x}}_{1},{\hat{x}}_{2},\hat{s}|x_{1},x_{2},y)$ such that the distortion constraints are satisfied, i.e., $Ed_{1}\left(X_{1},{\hat{X}}_{1}\right)\leq D_{1}$, $Ed_{2}\left(X_{2},{\hat{X}}_{2}\right)\leq D_{2}$, and $Ed_{s}^{\prime }\left(X_{1},\hat{S}\right)\leq D_{s}$. Let $p\left({\hat{x}}_{1},{\hat{x}}_{2},\hat{s}|y\right)=\sum_{x_{1},x_{2}}p\left(x_{1},x_{2}|y\right)p\left({\hat{x}}_{1},{\hat{x}}_{2},\hat{s}|x_{1},x_{2},y\right)$. Randomly and independently generate $2^{nR}$ sequence triples $\left({\hat{x}}_{1}^{n},{\hat{x}}_{2}^{n},{\hat{s}}^{n}\right)$ indexed by $m\in [1:2^{nR}]$, each according to $p({\hat{x}}_{1},{\hat{x}}_{2},\hat{s}|y)$. The whole codebook C, consisting of these sequence triples, is revealed to both the encoder and decoder. When observing the source messages $\left(x_{1}^{n},x_{2}^{n},y^{n}\right)$, find an index *m* such that its indexing sequence $\left({\hat{x}}_{1}^{n},{\hat{x}}_{2}^{n},{\hat{s}}^{n}\right)$ satisfies $\left(x_{1}^{n},x_{2}^{n},y^{n},{\hat{x}}_{1}^{n},{\hat{x}}_{2}^{n},{\hat{s}}^{n}\right)\in T_{\epsilon }^{n}$. If there is more than one such index, randomly choose one of them; if there is no such an index, set m=1. Upon receiving the index *m*, the decoder reconstructs the messages and inference by choosing the codeword $\left({\hat{x}}_{1}^{n},{\hat{x}}_{2}^{n},{\hat{s}}^{n}\right)$ indexed by *m*. By the law of large numbers, the source sequences are joint typical with probability 1 as n→∞. Then, we define the “encoding error” event as

(A1) $$E=\left\{\left(X_{1}^{n},X_{2}^{n},Y^{n},{\hat{X}}_{1}^{n},{\hat{X}}_{2}^{n},{\hat{S}}^{n}\right)\notin T_{\epsilon }^{n},\;\forall m\in \left[1:2^{nR}\right]\right\}.$$

Then, we can bound the error probability as follows

$$\begin{array}{cc} P(E)\; & =P\left\{\left(x_{1}^{n},x_{2}^{n},y^{n},{\hat{X}}_{1}^{n},{\hat{X}}_{2}^{n},{\hat{S}}^{n}\right)\notin T_{\epsilon }^{n},\;\forall m\in \left[1:2^{nR}\right]\right\}\\ & =\prod_{m=1}^{2^{nR}}P\left\{\left(x_{1}^{n},x_{2}^{n},y^{n},{\hat{X}}_{1}^{n},{\hat{X}}_{2}^{n},{\hat{S}}^{n}\right)\notin T_{\epsilon }^{n}\right\}\\ & ={\left(1-P\left\{\left(x_{1}^{n},x_{2}^{n},y^{n},{\hat{X}}_{1}^{n},{\hat{X}}_{2}^{n},{\hat{S}}^{n}\right)\in T_{\epsilon }^{n}\right\}\right)}^{2^{nR}}\\ & \leq \sum_{\left(x_{1}^{n},x_{2}^{n},y^{n}\right)\in T_{\epsilon }^{n}}\left[p\left(x_{1}^{n},x_{2}^{n},y^{n}\right)\cdot {\left(1-2^{-n[I\left(X_{1},X_{2};{\hat{X}}_{1},{\hat{X}}_{2},\hat{S}|Y\right)+\delta \left(\epsilon \right)]}\right)}^{2^{nR}}\right]\\ & \leq \exp\left(-2^{n[R-I\left(X_{1},X_{2};{\hat{X}}_{1},{\hat{X}}_{2},\hat{S}|Y\right)-\delta \left(\epsilon \right)]}\right), \end{array}$$

where δ(ϵ)→0 as n→∞, the first inequality follows from the joint typicality lemma in [22], and the last inequality follows from the fact that ${\left(1-z\right)}^{t}\leq \exp\left(-tz\right)$ for z∈[0,1] and t≥0. We see that P(E)→0 as n→∞ if $R>I\left(X_{1},X_{2};{\hat{X}}_{1},{\hat{X}}_{2},\hat{S}|Y\right)+\delta \left(\epsilon \right)$. If the error event does not happen, i.e., the reconstruction is joint typical with the source sequences, then from the distortion constraints assumed for the conditional pmf, the expected distortions can achieve $D_{1},D_{2},$ and $D_{s}$, respectively. This proves the achievability. Define $R_{I}\left(D_{1},D_{2},D_{s}\right)$ as the rate–distortion function characterized by Theorem 1. For the converse part, we show that

$$\begin{array}{cc} nR & \geq H\left(W\right)\geq H\left(W|Y^{n}\right)\geq I\left(X_{1}^{n},X_{2}^{n};W|Y^{n}\right)\\ & \geq I(X_{1}^{n},X_{2}^{n};{\hat{X}}_{1}^{n},{\hat{X}}_{2}^{n},{\hat{S}}^{n}|Y^{n})\\ & =I\left(X_{1}^{n},X_{2}^{n},Y^{n};{\hat{X}}_{1}^{n},{\hat{X}}_{2}^{n},{\hat{S}}^{n}\right)-I\left(Y^{n};{\hat{X}}_{1}^{n},{\hat{X}}_{2}^{n},{\hat{S}}^{n}\right)\\ & =\sum_{i=1}^{n}\left[I\left(X_{1,i},X_{2,i},Y_{i};{\hat{X}}_{1}^{n},{\hat{X}}_{2}^{n},{\hat{S}}^{n}|X_{1,1}^{i-1},X_{2,1}^{i-1},Y_{1}^{i-1}\right)-I\left(Y_{i};{\hat{X}}_{1}^{n},{\hat{X}}_{2}^{n},{\hat{S}}^{n}|Y_{1}^{i-1}\right)\right]\\ & =\sum_{i=1}^{n}\left[I\left(X_{1,i},X_{2,i},Y_{i};{\hat{X}}_{1}^{n},{\hat{X}}_{2}^{n},{\hat{S}}^{n},X_{1,1}^{i-1},X_{2,1}^{i-1},Y_{1}^{i-1}\right)-I\left(Y_{i};{\hat{X}}_{1}^{n},{\hat{X}}_{2}^{n},{\hat{S}}^{n},Y_{1}^{i-1}\right)\right]\\ & =\sum_{i=1}^{n}\left[I(X_{1,i},X_{2,i};{\hat{X}}_{1,i},{\hat{X}}_{2,i},{\hat{S}}_{i}|Y_{i})\right.\\ & \;\;\;\;\;\;+I(X_{1,i},X_{2,i},Y_{i};{\hat{X}}_{1,1}^{i-1},{\hat{X}}_{1,i+1}^{n},{\hat{X}}_{2,1}^{i-1},{\hat{X}}_{2,i+1}^{n},{\hat{S}}_{1}^{i-1},{\hat{S}}_{i+1}^{n},X_{1,1}^{i-1},X_{2,1}^{i-1},Y_{1}^{i-1}|{\hat{X}}_{1,i},{\hat{X}}_{2,i},{\hat{S}}_{i})\\ & \;\;\;\;\;\;-I(Y_{i};{\hat{X}}_{1,1}^{i-1},{\hat{X}}_{1,i+1}^{n},{\hat{X}}_{2,1}^{i-1},{\hat{X}}_{2,i+1}^{n},\left.{\hat{S}}_{1}^{i-1},{\hat{S}}_{i+1}^{n},Y_{1}^{i-1}\left|{\hat{X}}_{1,i},{\hat{X}}_{2,i},{\hat{S}}_{i}\right)\right]\\ & =\sum_{i=1}^{n}\left[I(X_{1,i},X_{2,i};{\hat{X}}_{1,i},{\hat{X}}_{2,i},{\hat{S}}_{i}|Y_{i})\right.+I\left(X_{1,i},X_{2,i},Y_{i};X_{1,1}^{i-1},X_{2,1}^{i-1}|{\hat{X}}_{1}^{n},{\hat{X}}_{2}^{n},{\hat{S}}^{n},Y_{1}^{i}\right)\\ & \;\;\;\;\;\;+I(X_{1,i},X_{2,i};{\hat{X}}_{1,1}^{i-1},{\hat{X}}_{1,i+1}^{n},{\hat{X}}_{2,1}^{i-1},{\hat{X}}_{2,i+1}^{n},\left.{\hat{S}}_{1}^{i-1},{\hat{S}}_{i+1}^{n},Y_{1}^{i-1}\left|{\hat{X}}_{1,i},{\hat{X}}_{2,i},{\hat{S}}_{i},Y_{i}\right)\right] \end{array}\;$$

(A2) $$\begin{array}{cc} & \geq \sum_{i=1}^{n}I\left(X_{1,i},X_{2,i};{\hat{X}}_{1,i},{\hat{X}}_{2,i},{\hat{S}}_{i}|Y_{i}\right) \end{array}\;$$

(A3) $$\begin{array}{cc} & \geq \sum_{i=1}^{n}R_{I}\left(Ed_{1}\left(X_{1,i},{\hat{X}}_{1,i}\right),Ed_{2}\left(X_{2,i},{\hat{X}}_{2,i}\right),Ed_{s}^{\prime }\left(X_{1,i},{\hat{S}}_{i}\right)\right) \end{array}\;$$

(A4) $$\begin{array}{cc} & \geq nR_{I}\left(Ed_{1}\left(X_{1}^{n},{\hat{X}}_{1}^{n}\right),Ed_{2}\left(X_{2}^{n},{\hat{X}}_{2}^{n}\right),Ed_{s}^{\prime }\left(X_{1}^{n},{\hat{S}}^{n}\right)\right) \end{array}\;$$

(A5) $$\begin{array}{cc} & \geq nR_{I}\left(Ed_{1}\left(X_{1}^{n},{\hat{X}}_{1}^{n}\right),Ed_{2}\left(X_{2}^{n},{\hat{X}}_{2}^{n}\right),Ed_{s}\left(S^{n},{\hat{S}}^{n}\right)\right) \end{array}\;$$

(A6) $$\begin{array}{cc} & \geq nR_{I}\left(D_{1},D_{2},D_{s}\right), \end{array}\;$$

where (A2) follows from the non-negativity of mutual information, (A3) follows from the definition of $R_{I}\left(D_{1},D_{2},D_{s}\right)$, (A4) follows from the convexity of $R_{I}\left(D_{1},D_{2},D_{s}\right)$, (A5) follows from $Ed_{s}^{\prime }\left(X_{1}^{n},{\hat{S}}^{n}\right)=Ed_{s}\left(S^{n},{\hat{S}}^{n}\right)$ which is proved in [11], and the last inequality follows from the non-increasing property of $R_{I}\left(D_{1},D_{2},D_{s}\right)$. This completes the converse proof. □

## Appendix B. Proof of Lemma 2

**Proof.** The Markov chain $X_{1}-Y-X_{2}$ indicates that

(A7) $$H\left(X_{2}|X_{1},Y\right)=H\left(X_{2}|Y\right).$$

Then, the mutual information in (9) can be bounded by

$$\begin{array}{cc} I(X_{1},X_{2};{\hat{X}}_{1},{\hat{X}}_{2},\hat{S}|Y)\; & =H\left(X_{1},X_{2}|Y\right)-H\left(X_{1},X_{2}|{\hat{X}}_{1},{\hat{X}}_{2},\hat{S},Y\right)\\ & =H\left(X_{1}|Y\right)+H\left(X_{2}|Y\right)-H\left(X_{1}|{\hat{X}}_{1},{\hat{X}}_{2},\hat{S},Y\right)-H\left(X_{2}|X_{1},{\hat{X}}_{1},{\hat{X}}_{2},\hat{S},Y\right)\\ & \geq H\left(X_{1}|Y\right)+H\left(X_{2}|Y\right)-H\left(X_{1}|{\hat{X}}_{1},\hat{S},Y\right)-H\left(X_{2}|{\hat{X}}_{2},Y\right)\\ & =I\left(X_{1};{\hat{X}}_{1},\hat{S}|Y\right)+I\left(X_{2};{\hat{X}}_{2}|Y\right), \end{array}$$

where the inequality follows from the fact that conditioning does not increase entropy. Now, we have

$$\begin{array}{cc} R(D_{1},D_{2},D_{s})\; & =\min_{\begin{array}{c} p({\hat{x}}_{1},{\hat{x}}_{2},\hat{s}|x_{1},x_{2},y)\\ Ed_{1}\left(X_{1},{\hat{X}}_{1}\right)\leq D_{1}\\ Ed_{2}\left(X_{2},{\hat{X}}_{2}\right)\leq D_{2}\\ Ed_{s}^{\prime }\left(X_{1},\hat{S}\right)\leq D_{s} \end{array}}I\left(X_{1},X_{2};{\hat{X}}_{1},{\hat{X}}_{2},\hat{S}|Y\right)\\ & \geq \min_{\begin{array}{c} p({\hat{x}}_{1},{\hat{x}}_{2},\hat{s}|x_{1},x_{2},y)\\ Ed_{1}\left(X_{1},{\hat{X}}_{1}\right)\leq D_{1}\\ Ed_{2}\left(X_{2},{\hat{X}}_{2}\right)\leq D_{2}\\ Ed_{s}^{\prime }\left(X_{1},\hat{S}\right)\leq D_{s} \end{array}}\left[I\left(X_{1};{\hat{X}}_{1},\hat{S}|Y\right)+I\left(X_{2};{\hat{X}}_{2}|Y\right)\right]\\ & =\min_{\begin{array}{c} p({\hat{x}}_{1},\hat{s}|x_{1},y)\\ Ed_{1}\left(X_{1},{\hat{X}}_{1}\right)\leq D_{1}\\ Ed_{s}^{\prime }\left(X_{1},\hat{S}\right)\leq D_{s} \end{array}}I\left(X_{1};{\hat{X}}_{1},\hat{S}|Y\right)+\min_{\begin{array}{c} p({\hat{x}}_{2}|x_{2},y)\\ Ed_{2}\left(X_{2},{\hat{X}}_{2}\right)\leq D_{2} \end{array}}I\left(X_{2};{\hat{X}}_{2}|Y\right)\\ & =R_{2d,X_{1}|Y}\left(D_{1},D_{s}\right)+R_{X_{2}|Y}\left(D_{2}\right). \end{array}$$

For the other direction, we show that the rate–distortion quadruple $(R_{2d,X_{1}|Y}\left(D_{1},D_{s}\right)+R_{X_{2}|Y}\left(D_{2}\right),$$D_{1},D_{2},D_{s})$ is achievable. To see this, let $p^{*}\left({\hat{x}}_{1},\hat{s}|x_{1},y\right)$ and $p^{*}\left({\hat{x}}_{2}|x_{2},y\right)$ be the optimal distributions that achieve the rate–distortion tuples $\left(R_{2d,X_{1}|Y}\left(D_{1},D_{s}\right),D_{1},D_{s}\right)$ and $\left(R_{X_{2}|Y}\left(D_{2}\right),D_{2}\right)$, respectively. Now, we consider the distribution $p^{*}\left(x_{1},x_{2},{\hat{x}}_{1},{\hat{x}}_{2},\hat{s}|y\right)≜p^{*}\left(x_{1},{\hat{x}}_{1},\hat{s}|y\right)p^{*}\left(x_{2},{\hat{x}}_{2}|y\right)$ which requires the Markov chain $\left(X_{1},{\hat{X}}_{1},\hat{S}\right)-Y-\left(X_{2},{\hat{X}}_{2}\right)$ and is consistent with the condition $X_{1}-Y-X_{2}$. Then, the corresponding random variables satisfy

$$\begin{array}{cc} I(X_{1},X_{2};{\hat{X}}_{1},{\hat{X}}_{2},\hat{S}|Y)\; & =H\left(X_{1}|Y\right)+H\left(X_{2}|Y\right)-H\left(X_{1}|{\hat{X}}_{1},{\hat{X}}_{2},\hat{S},Y\right)-H\left(X_{2}|X_{1},{\hat{X}}_{1},{\hat{X}}_{2},\hat{S},Y\right)\\ & =H\left(X_{1}|Y\right)+H\left(X_{2}|Y\right)-H\left(X_{1}|{\hat{X}}_{1},\hat{S},Y\right)-H\left(X_{2}|{\hat{X}}_{2},Y\right)\\ & =I\left(X_{1};{\hat{X}}_{1},\hat{S}|Y\right)+I\left(X_{2};{\hat{X}}_{2}|Y\right)\\ & =R_{2d,X_{1}|Y}\left(D_{1},D_{s}\right)+R_{X_{2}|Y}\left(D_{2}\right), \end{array}$$

where the first equality follows from (A7), the second equality follows from the Markov chain $\left(X_{1},{\hat{X}}_{1},\hat{S}\right)-Y-\left(X_{2},{\hat{X}}_{2}\right)$, and the last equality follows from the optimality of $p^{*}\left({\hat{x}}_{1},\hat{s}|x_{1},y\right)$ and $p^{*}\left({\hat{x}}_{2}|x_{2},y\right)$. Lastly, by the minimization in the expression of the rate–distortion function in (9), we conclude that $R\left(D_{1},D_{2},D_{s}\right)\leq R_{2d,X_{1}|Y}\left(D_{1},D_{s}\right)+R_{X_{2}|Y}\left(D_{2}\right)$, which completes the proof of the lemma. □

## Appendix C. Proof of Lemma 3

**Proof.** As we are in the binary Hamming setting, we first calculate $d_{s}^{\prime }\left(x_{1},\hat{s}\right)$ (c.f. (10)) by

$$\begin{array}{cc} d_{s}^{\prime }\left(0,0\right) & =\frac{1}{p(x_{1}=0)}\sum_{s=0,1}p\left(x_{1}=0,s\right)d_{s}\left(s,0\right)\\ & =\frac{1}{p(x_{1}=0)}\left[p\left(x_{1}=0,s=0\right)d_{s}\left(0,0\right)+p\left(x_{1}=0,s=1\right)d_{s}\left(1,0\right)\right]\\ & =\frac{p(x_{1}=0,s=1)}{p(x_{1}=0)}\\ & =p(s=1|x_{1}=0)=p. \end{array}$$

The other values follow similarly, and we obtain the distortion function

(A8) $$d_{s}^{\prime }\left(x_{1},\hat{s}\right)=\left\{\begin{array}{cc} p, & \mathrm{if}\;\hat{s}=x_{1}\\ 1-p, & \mathrm{if}\;\hat{s}\neq x_{1}. \end{array}\right.$$

Then,

$$\begin{array}{cc} Ed_{s}^{\prime }\left(X_{1},\hat{S}\right) & =\sum_{x_{1},\hat{s}}p\left(x_{1},\hat{s}\right)d_{s}^{\prime }\left(x_{1},\hat{s}\right)\\ & =P\left(X_{1}\neq \hat{S}\right)\cdot \left(1-p\right)+P\left(X_{1}=\hat{S}\right)\cdot p\\ & =P\left(X_{1}\neq \hat{S}\right)\left[1-2p\right]+p. \end{array}$$

The distortion constraint $Ed_{s}^{\prime }\left(X_{1},\hat{S}\right)\leq D_{s}$ for $D_{s}\geq p$ implies $P\left(X_{1}\neq \hat{S}\right)\leq \frac{D_{s}-p}{1-2p}$. Now, we can follow the rate–distortion evaluation for Bernoulli source and Hamming distortion in [23] while only changing the probability $P(X_{1}\neq \hat{S})$ and obtain

(A9) $$R_{s}\left(D_{s}\right)=\left[1-h_{b}\left(\frac{D_{s}-p}{1-2p}\right)\right]\cdot 1_{p\leq D_{s}\leq 0.5}.$$

This proves the lemma. □

## Appendix D. Proof of Theorem 3

**Proof.** Since Lemma 2 holds here, we first calculate the rate–distortion function for $X_{2}$, which is

(A10) $$\begin{array}{cc} R_{X_{2}|Y}\left(D_{2}\right) & =\left[1-h_{b}\left(D_{2}\right)\right]\cdot 1_{0\leq D_{2}\leq 0.5}. \end{array}$$

Now, it remains to calculate $R_{2d,X_{1}|Y}\left(D_{1},D_{s}\right)$. We first consider the case that $D_{1}\leq D_{s}^{0}$ and provide a lower bound of the mutual information as follows

$$\begin{array}{cc} I(X_{1};{\hat{X}}_{1},\hat{S}|Y)\; & \geq I\left(X_{1};{\hat{X}}_{1}|Y\right)=H\left(X_{1}|Y\right)-H\left(X_{1}|{\hat{X}}_{1},Y\right)\\ & \geq H\left(X_{1}|Y\right)-H\left(X_{1}|{\hat{X}}_{1}\right)\\ & =h_{b}\left(p_{2}\right)+\log\left(N/2\right)-H\left(X_{1}|{\hat{X}}_{1}\right)\\ & \geq h_{b}\left(p_{2}\right)+\log\left(N/2\right)-h_{b}\left(D_{1}\right)-D_{1}\log\left(N-1\right), \end{array}$$

where the last inequality follows from $P\left({\hat{X}}_{1}\neq X_{1}\right)\leq D_{1}$, $h_{b}\left(x\right)$ is an increasing function for x∈[0,0.5], and uniform distribution maximizes entropy. (Note that we can also obtain the above lower bound by directly applying the log-sum inequality.) Next, we show the lower bound is tight by finding a joint distribution that achieves the above rate and distortions $D_{1}$ and $D_{s}$. For $0\leq D_{1}\leq \frac{2\left(N-1\right)p_{2}}{N}$, we choose $\hat{S}={\hat{X}}_{1}$ and $\left(X_{1},Y,{\hat{X}}_{1}\right)$ by the test channel $p(x_{1}|{\hat{x}}_{1})$ in Figure A1 and the Markov chain $Y-{\hat{X}}_{1}-X_{1}$. The conditional probability of the test channel in Figure A1 is given as

(A11) $$\begin{array}{c} p\left({\hat{x}}_{1}|x\right)=\left\{\begin{array}{cc} 1-D_{1}, & \;\mathrm{if}\;{\hat{x}}_{1}=x\\ \frac{D_{1}}{N-1}, & \;\mathrm{if}\;{\hat{x}}_{1}\neq x. \end{array}\right. \end{array}$$

Solving the equations

$$\begin{array}{c} q_{i}\left(1-D_{1}\right)+\left(\sum_{j\in [1:N],j\neq i}q_{j}\right)\frac{D_{1}}{N-1}=\frac{1}{N},\;i\in \left[1:N\right], \end{array}$$

we obtain that $q_{i}=\frac{1}{N}$ for i∈[1:N], i.e., ${\hat{X}}_{1}$ is also uniformly distributed over [1:N]. For the joint distribution of *Y* and ${\hat{X}}_{1}$, we define

p(y|x^1)=p2−ND12(N−1)1−ND1N−1,if y=0,x^1 is odd   or y=1,x^1 is even  1−p2−ND12(N−1)1−ND1N−1,if y=0,x^1 is even   or y=1,x^1 is odd.

We see that $p(y|{\hat{x}}_{1})\geq 0$ for any $D_{1}\leq \frac{2\left(N-1\right)p_{2}}{N}$, i.e., $\left(D_{1},D_{2},D_{s}\right)\in D_{1}$. Then, we can verify using $p\left(y|x_{1}\right)=\sum_{{\hat{x}}_{1}}p\left(y|x_{1},{\hat{x}}_{1}\right)p\left(\hat{x}|x\right)=\sum_{{\hat{x}}_{1}}p\left(y|{\hat{x}}_{1}\right)p\left(\hat{x}|x\right)$ that the above distribution can induce the same conditional probability $p(y|x_{1})$ (with transition probability $p_{2}$) as defined at the beginning of this section. Thus, we have constructed a feasible $p(x_{1},y,{\hat{x}}_{1})$ that can achieve expected distortion $Ed_{1}\left(X,{\hat{X}}_{1}\right)=D_{1}$, $D_{s}^{0}\geq D_{1}$, and mutual information

$$\begin{array}{cc} I(X_{1};{\hat{X}}_{1},\hat{S}|Y)\; & =I\left(X_{1};{\hat{X}}_{1}|Y\right)=H\left(X_{1}|Y\right)-H\left(X_{1}|{\hat{X}}_{1},Y\right)\\ & =H\left(X_{1}|Y\right)-H\left(X_{1}|{\hat{X}}_{1}\right)\\ & =h_{b}\left(p_{2}\right)+\log\left(N/2\right)-h_{b}\left(D_{1}\right)-D_{1}\log\left(N-1\right), \end{array}$$

where the first equality follows from $\hat{S}={\hat{X}}_{1}$, the third equality follows from the Markov chain $Y-{\hat{X}}_{1}-X_{1}$, and the last equality follows from the joint distribution of $\left(X_{1},Y\right)$ and the distribution in (A11). For the other case that $D_{1}\geq D_{s}^{0}$, we only need to switch the role of $\left({\hat{X}}_{1},D_{1}\right)$ and $\left(\hat{S},D_{s}^{0}\right)$. Then, the rate and distortions can be obtained accordingly, which can prove the theorem. □

## Appendix E. Proof of Theorem 4

**Proof.** The rate–distortion function in Theorem 1 satisfies

(A12) $$R\left(D_{1},D_{2},D_{s}\right)=R_{2d,X_{1}|Y}\left(D_{1},D_{s}\right)+R_{X_{2}|Y}\left(D_{2}\right).$$

The second term is the solution to the quadratic Gaussian source coding problem with side information [22] [Chapter 11], which is given as

(A13) $$R_{X_{2}|Y}\left(D_{2}\right)=\frac{1}{2}{\left(\log\frac{\sigma_{X_{2}|Y}}{D_{2}}\right)}^{+},$$

where $\sigma_{X_{2}|Y}$ is the conditional variance of $X_{2}$ given *Y*. Note that $X_{2}$ is Gaussian conditioning on *Y*, i.e., $X_{2}|Y∼N\left(\frac{\sigma_{X_{2}Y}}{\sigma_{Y}}Y,\sigma_{X_{2}}-\frac{\sigma_{X_{2}Y}^{2}}{\sigma_{Y}}\right)$, which implies $\sigma_{X_{2}|Y}=\sigma_{X_{2}}-\frac{\sigma_{X_{2}Y}^{2}}{\sigma_{Y}}$. For the first term, note that $R_{2d,X_{1}|Y}\left(D_{1},D_{s}\right)$ is lower bounded by both

(A14) $$R_{X_{1}|Y}\left(D_{1}\right)=\min_{Ed_{1}\left(X_{1},{\hat{X}}_{1}\right)\leq D_{1}}I\left(X_{1};{\hat{X}}_{1}|Y\right),$$

and

(A15) $$R_{S|Y}\left(D_{s}\right)=\min_{Ed_{S}^{\prime }\left(X_{1},\hat{S}\right)\leq D_{s}}I\left(X_{1};\hat{S}|Y\right).$$

Obviously, (A14) is the solution of the quadratic Gaussian source coding with side information similarly to (A13). We proceed to calculate (A15), which is actually the semantic rate–distortion function of the indirect source coding with side information. Observing $\left(X_{1},Y\right)$, *S* is conditionally Gaussian as $S|\left(X_{1},Y\right)∼N\left(\frac{\sigma_{SX_{1}}}{\sigma_{X_{1}}}X_{1},\sigma_{S}-\frac{\sigma_{SX_{1}}^{2}}{\sigma_{X_{1}}}\right)$. It is shown in [29] that we can rewrite the semantic distortion as

(A16) $$Ed_{S}^{\prime }\left(X_{1},\hat{S}\right)=E\left[{\left(S-{\tilde{S}}_{\mathrm{MMSE}}\right)}^{2}\right]+E\left[{\left({\tilde{S}}_{\mathrm{MMSE}}-\hat{S}\right)}^{2}\right],$$

where ${\tilde{S}}_{\mathrm{MMSE}}=\frac{\sigma_{SX_{1}}}{\sigma_{X_{1}}}X_{1}$ is the MMSE estimator upon observing $X_{1}$ and *Y*, and the first term on the right-hand side is the corresponding minimum mean squared error ($D_{\mathrm{MMSE}}$, c.f. (18)), i.e.,

(A17) $$E\left[{\left(S-{\tilde{S}}_{\mathrm{MMSE}}\right)}^{2}\right]=D_{\mathrm{MMSE}}=\sigma_{S}-\frac{\sigma_{SX_{1}}^{2}}{\sigma_{X_{1}}}.$$

Then, we consider a specific encoder which first estimates the semantic information using the MMSE estimator and then compresses the estimation under mean squared error distortion constraint $D_{s}-D_{\mathrm{MMSE}}$ with side information. The resulting achievable rate provides an upper bound on $R_{S|Y}\left(D_{s}\right)$, i.e.,

$$\begin{array}{cc} R_{S|Y}\left(D_{s}\right) & \leq \frac{1}{2}{\left(\log\frac{\sigma_{{\tilde{S}}_{\mathrm{MMSE}}|Y}}{D_{s}-D_{\mathrm{MMSE}}}\right)}^{+}\\ & =\frac{1}{2}{\left(\log\frac{\sigma_{SX_{1}}^{2}\sigma_{X_{1}|Y}}{\sigma_{X_{1}}^{2}\left(D_{s}-D_{\mathrm{MMSE}}\right)}\right)}^{+} \end{array}\;$$

(A18) $$\begin{array}{cc} & =\frac{1}{2}{\left(\log\frac{\sigma_{SX_{1}}^{2}\left(\sigma_{X_{1}}-\frac{\sigma_{X_{1}Y}^{2}}{\sigma_{Y}}\right)}{\sigma_{X_{1}}^{2}\left(D_{s}-D_{\mathrm{MMSE}}\right)}\right)}^{+}. \end{array}$$

Furthermore, for $D_{s}\geq D_{\mathrm{MMSE}}+\frac{\sigma_{SX_{1}}^{2}\sigma_{X_{1}|Y}}{\sigma_{X_{1}}^{2}}$, we have $R_{S|Y}\left(D_{s}\right)=0$, which can be obtained by setting $\hat{S}=E\left[{\tilde{S}}_{\mathrm{MMSE}}|Y\right]=\frac{\sigma_{SX_{1}}}{\sigma_{X_{1}}}E\left[X_{1}|Y\right]$. For $D_{s}<D_{\mathrm{MMSE}}+\frac{\sigma_{SX_{1}}^{2}\sigma_{X_{1}|Y}}{\sigma_{X_{1}}^{2}}$, we derive a lower bound for $R_{S|Y}\left(D_{s}\right)$ as follows

$$\begin{array}{cc} R_{S|Y}\left(D_{s}\right) & \geq I\left(X_{1};\hat{S}|Y\right)=H\left(X_{1}|Y\right)-H\left(X_{1}|\hat{S},Y\right)\\ & =\frac{1}{2}\log\left(2\pi e\sigma_{X_{1}|Y}\right)-H\left(X_{1}-\frac{\sigma_{X_{1}}}{\sigma_{SX_{1}}}\hat{S}|\hat{S},Y\right)\\ & \geq \frac{1}{2}\log\left(2\pi e\sigma_{X_{1}|Y}\right)-H\left(X_{1}-\frac{\sigma_{X_{1}}}{\sigma_{SX_{1}}}\hat{S}\right) \end{array}\;$$

(A19) $$\begin{array}{cc} & \geq \frac{1}{2}\log\left(2\pi e\sigma_{X_{1}|Y}\right)-\frac{1}{2}\log\left(2\pi eE\left[{\left(X_{1}-\frac{\sigma_{X_{1}}}{\sigma_{SX_{1}}}\hat{S}\right)}^{2}\right]\right) \end{array}\;$$

(A20) $$\begin{array}{cc} & \geq \frac{1}{2}\log\left(2\pi e\sigma_{X_{1}|Y}\right)-\frac{1}{2}\log\left(2\pi e\frac{\sigma_{X_{1}}^{2}\left(D_{s}-D_{\mathrm{MMSE}}\right)}{\sigma_{SX_{1}}^{2}}\right) \end{array}\;$$

(A21) $$\begin{array}{cc} & =\frac{1}{2}\log\frac{\sigma_{SX_{1}}^{2}\sigma_{X_{1}|Y}}{\sigma_{X_{1}}^{2}\left(D_{s}-D_{\mathrm{MMSE}}\right)}=\frac{1}{2}\log\frac{\sigma_{SX_{1}}^{2}\left(\sigma_{X_{1}}-\frac{\sigma_{X_{1}Y}^{2}}{\sigma_{Y}}\right)}{\sigma_{X_{1}}^{2}\left(D_{s}-D_{\mathrm{MMSE}}\right)}, \end{array}$$

where (A19) is due to the fact that the Gaussian distribution maximizes the entropy for a given variance, and (A20) follows from (A16) and the semantic distortion constraint. Combining the upper and lower bounds in (A18) and (A21), we obtain

(A22) $$R_{S|Y}\left(D_{s}\right)=\frac{1}{2}{\left(\log\frac{\sigma_{SX_{1}}^{2}\left(\sigma_{X_{1}}-\frac{\sigma_{X_{1}Y}^{2}}{\sigma_{Y}}\right)}{\sigma_{X_{1}}^{2}\left(D_{s}-D_{\mathrm{MMSE}}\right)}\right)}^{+}.$$

Thus, we have

(A23) $$\begin{array}{c} \begin{array}{cc} R(D_{1}, & D_{2},D_{s})\geq \max\left\{R_{X_{1}|Y}\left(D_{1}\right),R_{S|Y}\left(D_{s}\right)\right\}+R_{X_{2}|Y}\left(D_{2}\right)\\ & =\frac{1}{2}{\left(\log\frac{\sigma_{X_{2}}-\frac{\sigma_{X_{2}Y}^{2}}{\sigma_{Y}}}{D_{2}}\right)}^{+}+\frac{1}{2}{\left[\log\max\left(\frac{\sigma_{X_{1}}-\frac{\sigma_{X_{1}Y}^{2}}{\sigma_{Y}}}{D_{1}},\frac{\sigma_{SX_{1}}^{2}\left(\sigma_{X_{1}}-\frac{\sigma_{X_{1}Y}^{2}}{\sigma_{Y}}\right)}{\sigma_{X_{1}}^{2}\left(D_{s}-D_{\mathrm{MMSE}}\right)}\right)\right]}^{+}. \end{array} \end{array}$$

To show the achievability, consider the following two cases.

- For $\frac{D_{s}-D_{\mathrm{MMSE}}}{\sigma_{SX_{1}}^{2}}\geq \frac{D_{1}}{\sigma_{X_{1}}^{2}}$, we first reconstruct ${\hat{X}}_{1}$ and $X_{2}$ subject to distortion constraints $D_{1}$ and $D_{2}$ and hence achieve $R_{X_{1}|Y}\left(D_{1}\right)+R_{X_{2}|Y}\left(D_{2}\right)$. Then, we recover the semantic information by $\hat{S}=\frac{\sigma_{SX_{1}}}{\sigma_{X_{1}}}{\hat{X}}_{1}$, and the semantic distortion satisfies
  $$\begin{array}{c} Ed_{S}^{\prime }\left(X_{1},\hat{S}\right)=D_{\mathrm{MMSE}}+E\left[{\left(\frac{\sigma_{SX_{1}}}{\sigma_{X_{1}}}X_{1}-\frac{\sigma_{SX_{1}}}{\sigma_{X_{1}}}{\hat{X}}_{1}\right)}^{2}\right]\leq D_{\mathrm{MMSE}}+\frac{\sigma_{SX_{1}}^{2}}{\sigma_{X_{1}}^{2}}D_{1}\leq D_{s}. \end{array}$$
- For $\frac{D_{s}-D_{\mathrm{MMSE}}}{\sigma_{SX_{1}}^{2}}<\frac{D_{1}}{\sigma_{X_{1}}^{2}}$, we first reconstruct $\hat{S}$ and $X_{2}$ subject to distortion constraints $D_{s}$ and $D_{2}$ and hence achieve $R_{S|Y}\left(D_{s}\right)+R_{X_{2}|Y}\left(D_{2}\right)$. Then, we recover ${\hat{X}}_{1}=\frac{\sigma_{X_{1}}}{\sigma_{SX_{1}}}\hat{S}$, and the distortion satisfies
  $$\begin{array}{c} Ed_{1}\left(X_{1},{\hat{X}}_{1}\right)=E\left[{\left(X_{1}-\frac{\sigma_{X_{1}}}{\sigma_{SX_{1}}}\hat{S}\right)}^{2}\right]=\frac{\sigma_{X_{1}}^{2}}{\sigma_{SX_{1}}^{2}}\left(Ed_{S}^{\prime }\left(X_{1},\hat{S}\right)-D_{\mathrm{MMSE}}\right)<D_{1}. \end{array}$$

This establishes the achievability and thus completes the proof. □
